# Vocal cord palsy: An uncommon presenting feature of myasthenia gravis

**DOI:** 10.4103/0972-2327.78049

**Published:** 2011

**Authors:** Prahlad K. Sethi, Anuradha Batra, Nitin K. Sethi, Josh Torgovnick, Edward Arsura

**Affiliations:** Department of Neurology, Sir Ganga Ram Hospital, New Delhi, India; 1 Department of Neurology, New York-Presbyterian Hospital, Weill Cornell Medical Center, New York, NY, USA; 2 Department of Neurology, Saint Vincent’s Hospital and Medical Centers, New York, NY, USA; 3 Department of Medicine, Saint Vincent’s Hospital and Medical Centers, New York, NY, USA

**Keywords:** Myasthenia gravis, respiratory distress, stridor, vocal cord palsy, vocal cord paralysis

## Abstract

Vocal cord palsy can have myriad causes. Unilateral vocal cord palsy is common and frequently asymptomatic. Trauma, head, neck and mediastinal tumors as well as cerebrovascular accidents have been implicated in causing unilateral vocal cord palsy. Viral neuronitis accounts for most idiopathic cases. Bilateral vocal cord palsy, on the other hand, is much less common and is a potentially life-threatening condition. Myasthenia gravis, an autoimmune disorder caused by antibodies targeting the post-synaptic acetylcholine receptor, has been infrequently implicated in its causation. We report here a case of bilateral vocal cord palsy developing in a 68-year-old man with no prior history of myasthenia gravis 2 months after he was operated on for diverticulitis of the large intestine. Delay in considering the diagnosis led to endotracheal intubation and prolonged mechanical ventilation with attendant complications. Our case adds to the existing literature implicating myasthenia gravis as an infrequent cause of bilateral vocal cord palsy. Our case is unusual as, in our patient, acute-onset respiratory distress and stridor due to bilateral vocal cord palsy was the first manifestation of a myasthenic syndrome.

## Introduction

Myasthenia gravis has been infrequently implicated in the causation of bilateral vocal cord palsy, presenting clinically with acute respiratory distress. In the majority of the cases reported in the literature, delay in considering the diagnosis led to either endotracheal intubation and prolonged mechanical ventilation or emergency tracheostomy with attendant complications.

## Case Report

A-68-year-old male with no significant past medical history presented to our hospital with acute respiratory distress and inspiratory stridor, necessitating emergency intubation and mechanical ventilation. Two months earlier, he had been operated upon in our hospital for diverticulitis of the large intestine and had made an uneventful recovery. In the intensive care unit, fiberoptic laryngoscopy carried out to determine the cause of his stridor revealed immobile vocal cords in the paramedian position. Neurology consultation was requested to rule out the possibility of vocal cord paralysis due to involvement of the lower cranial nerves. Magnetic resonance imaging of the brain and spinal cord was normal and cerebrospinal fluid studies excluded infectious or inflammatory pathology. Tracheostomy was under consideration to ease the management of secretions. The next day, while been re-examined by one of us (PKS), mild drooping of the left eyelid was noticed with positive fatigability. Neurological examination was limited as the patient was intubated, but there was no definite clinical weakness of the proximal musculature and forward arm abduction time was within normal limits (power 5/5 medical research council grade). We were unable to clinically assess the bulbar musculature and muscles of mastication on account of the patient’s intubated state. To identify myasthenia gravis, the Tensilon (edrophonium) test was performed, which briefly relieved eyelid weakness. During this procedure, the patient was carefully monitored because edrophonium can cause significant bradycardia, heart block and asystole. Therefore, atropine and cardiopulmonary resuscitation equipment was made available at the bedside. Single-fiber electromyography and repetitive nerve stimulation (RNS) studies to assess for increased jitter and a decremental response, respectively, were not performed as they were unavailable on an emergent basis. Injection neostigmine 3 mg intravenously under continuous cardiac monitoring every 6-hourly was initiated and, within 48 h, the patient was able to be weaned off the ventilator. The total dose administered was 24 mg over 48 h. Acetylcholine receptor antibodies (anti-AChR) came back negative. Anti-MuSK antibodies were not sent due to the non-availability of a testing facility.

## Discussion

Respiratory impairment in myasthenia gravis is usually on account of weakness of the diaphragm and intercostal muscles that move the thoracic cage. Functional upper airway obstruction due to vocal cord paralysis is a rarely reported manifestation of myasthenia gravis. Colp *et al*. described a 44-year-old white woman who immediately, after surgery, developed respiratory stridor apparently caused by bilateral vocal cord paralysis. Her first manifestation of the myasthenic syndrome was unmasked following exposure to a curare type of muscle relaxant during surgery. Over the next few days, their patient developed a more classical myasthenic manifestation with weakness of both extremity and respiratory muscles.[1] The unmasking of myasthenia gravis following the use of curare-type neuromuscular blockers during surgery has been reported before in the literature. Depolarizing neuromuscular blockers act by producing sustained depolarization of the motor endplate and their use in a patient with latent myasthenia may thus precipitate acute respiratory distress and stridor (myasthenic crises). Our patient had been operated upon for diverticulitis of the large intestine 2 months prior to his current presentation with an uneventful full recovery. Hence, in our patient, we cannot attribute the unmasking of myasthenia gravis to potential exposure to a curare-type muscle relaxant during abdominal surgery. Hara *et al*. reported a 56-year-old man who presented with a 3-month history of exertional dyspnea and stridor. Fiberoptic laryngoscopy showed paramedian position of the vocal cords with impaired abduction in inspiration. Vocal cord position did not change after an intravenous injection of edrophonium. Their patient however had presented earlier with proximal muscle weakness and atrophy and the RNS study had revealed a decremental response. In their patient, anti-AChR antibodies were not detected but elevated anti-MuSK antibodies were found.[2] Schmidt-Nowara *et al*. reported the case of a 63-year-old previously healthy man who had repeated paroxysms of coughing, choking, intense dyspnea, apnea and cyanosis. Breathing was stridorous and myasthenia gravis was correctly diagnosed when fatigue with chewing and intermittent slurring of speech developed.[3]

Functional upper airway obstruction presenting as stridor may not be attributed to myasthenia gravis unless a high index of clinical suspicion is maintained. This is especially true when it is the only and the first manifestation of myasthenia gravis, as was the case with our patient. Conventional spirometry, which measures expiratory function only, will not detect the upper airway obstruction. However, forced inspiration may produce acute airway occlusion, manifesting as stridor and paroxysms of coughing, choking, dyspnea and cyanosis. Hence, if myasthenia gravis is suspected as the cause of upper airway obstruction, a flow-volume loop should be used to clinically follow these patients.[3]

Dysphagia and dysphonia are the most frequently reported otolaryngological manifestations of myasthenia gravis. Bilateral vocal cord abductor paralysis due to selective involvement of the muscles of the larynx is commonly reported in patients with multiple system atrophy, including Shy-Drager syndrome.[4] Our case is unusual as, in our patient, acute-onset respiratory distress and stridor due to bilateral vocal cord palsy was the first manifestation of a myasthenic syndrome and he was not exposed to the curare group of muscle relaxants. Mechanistically, a muscle that is required to perform in an extraordinary fashion (post-intubation stressed cricoarytenoid muscles) is more likely to experience fatigue.

## Conclusion

It is important to consider myasthenia gravis in the differential diagnosis of bilateral vocal cord palsy that may present clinically as stridor. It is also important to remember that in some patients this may be the first and only manifestation of the myasthenic syndrome. Prompt diagnosis and appropriate treatment shall avoid the need for emergency tracheostomy.
